# The Effects of the Solution-Focused Model on Anxiety and Postpartum Depression in Nulliparous Pregnant Women

**DOI:** 10.3389/fpsyg.2022.814892

**Published:** 2022-04-04

**Authors:** Cuiqin Huang, Wei Han, Sanlian Hu

**Affiliations:** ^1^Department of Obstetrics, Shanghai Jiao Tong University Affiliated Sixth People’s Hospital, Shanghai, China; ^2^Nursing Department, Shanghai Jiao Tong University Affiliated Sixth People’s Hospital, Shanghai, China

**Keywords:** solution-focused model, anxiety, postpartum depression, nulliparous, pregnant

## Abstract

**Background:**

Solution-focused model (SFM) is an intervention method that fully mobilizes patients’ initiative through their potential. We aimed to investigate the effects of SFM on anxiety and postpartum depression (PPD) in nulliparous pregnant women compared with routine care services.

**Methods:**

We chose the mothers diagnosed as depressed or with depressive tendency by Edinburgh Postpartum Depression Scale (EPDS) at 28 weeks of gestation and divided them into the intervention and control groups. The control group only took the routine pregnancy healthy nursing, while the SFM group took the regular nursing and SFM counselling. Different assessments were conducted at 28 weeks of gestation, post-delivery, and post-intervention to evaluate the anxiety and depression levels of the patients. Finally, nursing satisfaction was evaluated by the nursing satisfaction questionnaire.

**Results:**

Compared with the control group, SFM could decrease the scores of anxiety and depression more effectively and influence sleep quality more positively. We also found that SFM resulted in significantly higher nursing satisfaction than that in the control group (*p* = 0.0046).

**Conclusion:**

In conclusion, SFM could effectively alleviate anxiety and PPD in nulliparous pregnant women.

## Introduction

Pregnancy is a crucial and unique physiological period in women’s life. During pregnancy, women undergo complex changes in physiology and experience intense stress reactions in psychology. Maternal postpartum depression (PPD) is a common and severe mental disorder that adversely affects mothers and infants’ health and the quality of postnatal attachment bond (Letourneau et al., 2012; Ponti et al., 2020). Furthermore, it could compromise mothers’ family roles, even leading to severe cases, such as suicide or infanticide (Sit et al., 2011). The average prevalence of PPD was 17% among mothers globally (Shorey et al., 2018). Nulliparous women become a vulnerable population because it is difficult to distinguish between the reality of first-time motherhood and the symptoms of PPD (Gold, 2002). Nulliparous women are not fully aware of the emotional fluctuations caused by hormone changes during pregnancy, and are not fully prepared for the weight and body changes during pregnancy. They lack confidence and preparation in raising children. On the other hand, multiparous women are different. They have more experience in pregnancy, childbirth, and raising children, so they are better prepared psychologically.

The psychological nursing of PPD is always focused on the negative emotions of the mothers, which makes pregnant women lack initiative and they sometimes cannot actively participate in it (Mcallister, 2003). For preventing PPD in women, a variety of psychosocial and psychological interventions have been developed, including home visit, interpersonal psychotherapy, and telehealth support (Dennis and Dowswell, 2013). Compared with these interventions, the solution-focused model (SFM), also called the solution-focused approach, is a different intervention method that focuses on solutions rather than the problems, which fully mobilizes patients’ subjective initiative using patients’ own abilities (Mcallister, 2003). Steve de Shazer developed SFM in the early 1980s, emphasizing that patients focus on the positive aspects of problem-solving to maximize their strength and potential (De Shazer et al., 1986). The SFM has been established in a professional psychotherapeutic application and clinical application and gained positive outcomes (Gonzalez, 2017; Gan, 2020; Woodger et al., 2021). Therefore, rather than passively facing problems, we thought that SFM could better guide patients to resolve their destructive emotions actively after giving birth so that nulliparous pregnant women can live a more active life. In addition, there were different degrees of sleep problems and psychological stress in pregnant and parturient women due to the lack of pregnancy and delivery experience. Previous study showed that alleviating sleep problems helped attenuate mental disorders and keep patients away from depression (Cuijpers, 2011). Therefore, the test of sleep quality could be an evaluation criterion of PPD.

This study was designed to help mothers acquire abilities to relieve the symptoms of PPD and then improve the health of mothers and their families. In order to control the variables, we selected more suitable objects for the study, namely nulliparous women. The purpose of this study was to investigate the effects of SFM on anxiety and PPD in nulliparous pregnant women compared with routine care services.

## Materials and Methods

### Inclusion and Exclusion Criteria

Inclusion criteria: (1) nulliparous pregnant women with singleton pregnancy; (2) no obstetric diseases such as placenta previa, eclampsia, or premature rupture of membranes; (3) no language barriers and good communication skills; (4) Edinburgh Postpartum Depression Scale (EPDS) score greater than nine; and (5) the participants signed informed consent.

Exclusion criteria: (1) EPDS score less than nine; (2) previous history of heart, lung, liver, kidney, and other serious basic diseases; (3) gestational diabetes, hypertension, or autoimmune diseases; (4) previous history of mental illness; and (5) malformed fetus.

### Solution-Focused Model

The SFM counselling typically consisted of five parts: describing the problem, developing well-formed goals, exploring exceptions, ending session feedback, and evaluating results. According to previous study, SFM intervention can achieve significant results after 3–5 times (Stams et al., 2006).

#### Describing the Problem

Firstly, we encouraged pregnant women to express their problems and discussed the problems with pregnant women and their families. Then, we enhanced their confidence in solving the issues. When similar issues occurred again, they could know how to deal with them. Finally, we tried to tap their problem-solving potential and help them ameliorate depression and anxiety.

#### Developing Well-Formed Goals

Pregnant women were encouraged to establish specific and feasible goals based on their conditions. Then, we worked with them to find solutions to the problems.

#### Exploring for Exceptions

After setting clear objectives, we continued to discuss the situations when the problems did not occur or similar problems were not seriously or accidentally resolved. Therefore, the pregnant women learned that their unconscious efforts might have changed some problems. At the same time, they discovered that their unconscious efforts could not be ignored even small changes occurred. These unconscious efforts could prompt their further thinking about making those exceptions happen again. Detection of exception was the focus of the SFM, which mainly made the pregnant women define their value, find changes, and solve problems. We also helped pregnant women establish their confidence to solve their problems and achieve their goals.

#### End of Session Feedback

Based on the previous communication and understanding, we tried to explore the advantages, resources, and efforts of the pregnant women and timely praised them with positive feedback. It enhanced the possibility of achieving the goals. If the pregnant women did not achieve the expected goals, we would discuss the reasons with them in time, adjust the expected goals according to the specific situation, and share the successful experience of other pregnant women with them so that they had more understanding of the method and improved their confidence to solve the problem in future. We mainly used the graduated questions to refine the achievement of goals and the direction of goals for pregnant women.

#### Evaluation Effect

At this stage, we continued to use the graduated questions to emphasize the importance of change to pregnant women, starting from small change and gradually increasing the degree of change. Pregnant women should be encouraged in time to help them build confidence and realize their efforts to solve problems.

The sequence of the five steps mentioned above was not completely fixed but flexible in the intervention process, guiding the pregnant women to achieve their goals. Therefore, pregnant women could successfully solve similar problems in the future.

### Design and Participants

**Figure 1:**
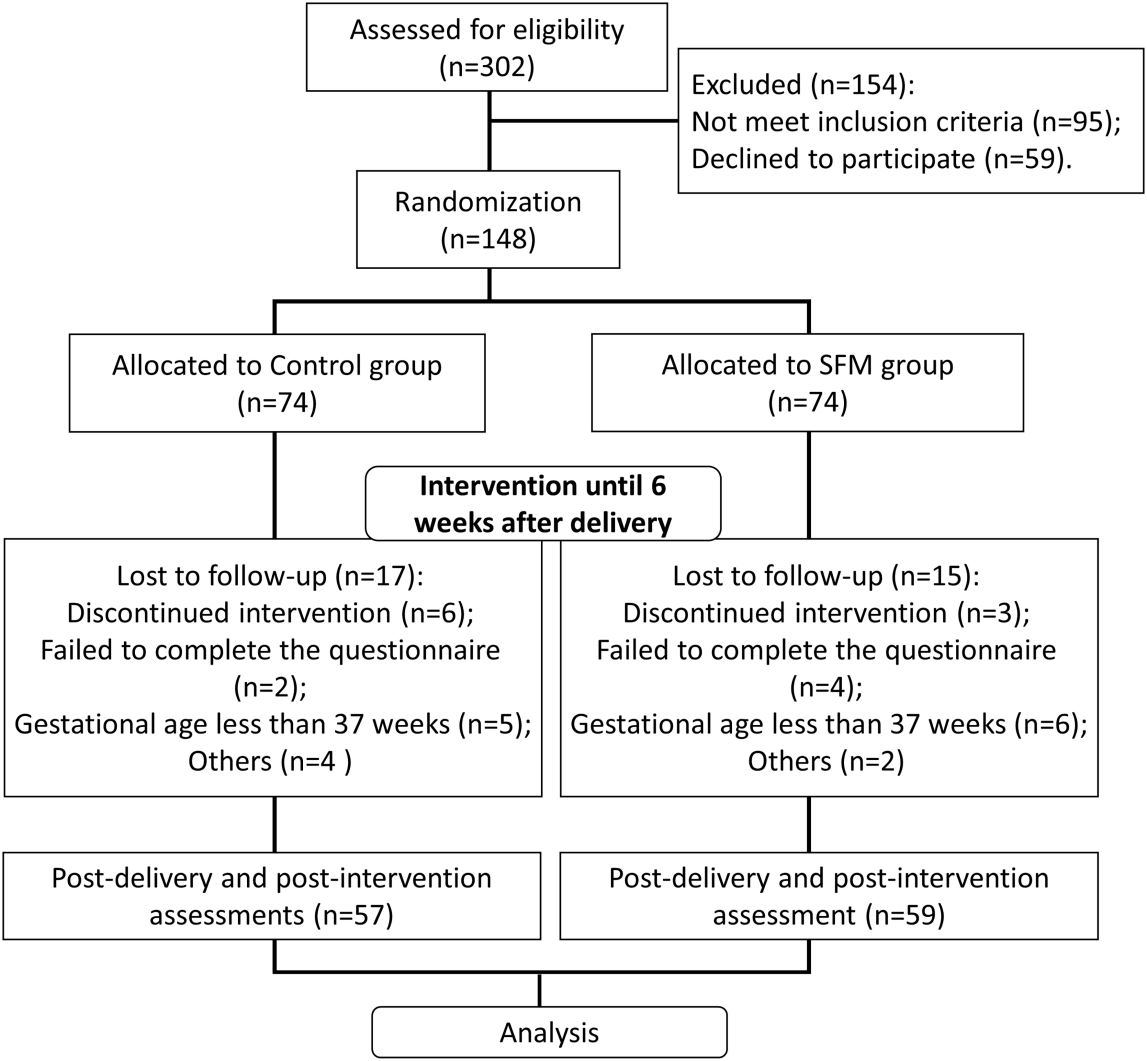
Research framework of this study. SFM, solution-focused model.

Figure 1 shows the flowchart of our study. In our study, 302 eligible pregnant women at 28 weeks of gestation were enrolled. According to the inclusion criteria, 95 pregnant women were excluded. After qualification of case evaluation, our physician team briefly introduced the knowledge and potential adverse effect of PPD to the pregnant women, informed them that we were conducting a nursing study to help them ameliorate depression and asked the pregnant women whether they would like to participate in our study. A total of 59 pregnant women declined to participate. Finally, 148 participants were recruited and randomly divided into the intervention group (the SFM group) and the control group, with 74 patients in each group. The study was a double-blind trial, and the patients in the two groups were not informed about their grouping and the way of care.

The basic demographic characteristics of all the participants were recorded, including age, education status, and occupation. This research was approved by the Ethical Committee of Shanghai Jiao Tong University Affiliated Sixth People’s Hospital. Informed consent was obtained from all participants.

### Intervention

Prenatal psychological interventions were performed from 28 weeks of gestation to 6 weeks after delivery. The control group received routine pregnancy health care service without any counselling. The intervention group took both the SFM counselling and routine care service. The routine care service included general symptom nursing, daily schedule guidance, diet nursing, and health education. The content of routine health education included routine pregnancy check, medication during pregnancy, prenatal ultrasound, prenatal fitness, nutrition during pregnancy, weight control during pregnancy, fetal growth and development process, scientific antenatal training, choice of delivery mode, delivery signs, breastfeeding, newborn care, and puerperium care. In the process of health education, researchers gave specific health guidance according to the needs of pregnant women. For the SFM group, prenatal counselling was mainly based on the check-up time of fetal heart monitoring (once a week from 30 to 39 weeks of gestation, five times in total). Postpartum counselling was carried out when the puerpera had a good condition without affecting breastfeeding or rest time (once at hospitalization, three times after discharge). The intervention process was one-on-one. Each counselling last 30–60 min. When the puerpera returned home, the intervention was conducted once every 2 weeks mainly at the puerpera’s home. The professional trainee conducted the counselling. The interventions took place in the fetal monitor room before pregnancy, in the ward during postpartum hospitalization, and at home after discharge.

Each part of the SFM represented a part of the whole intervention, and everyone had personalized path. We first asked pregnant women to describe the problem, and then each pregnant woman would have their own unique difficulties. Then, the nursing team would develop well-formed goals and explore for exceptions and feedback accordingly.

After the intervention, we had some patients withdrew in both groups due to loss of connection, discontinued intervention, or failure to complete the questionnaire. We also set the criteria of giving up midway: (1) gestational age less than 37 weeks; (2) interruption of intervention due to significant personal or family events that seriously affect pregnancy outcome or maternal physical and mental health; and (3) postpartum complications. Finally, 57 patients were left in the control group, while 59 patients were left in the SFM group.

### Outcome Measure

The primary outcome was self-reported depressive symptoms as measured by the Edinburgh Postnatal Depression Scale (EPDS). The EPDS contains 10 self-rating items (Cox et al., 1987). Each item was scored by four grades, with 0 for never, 1 for occasionally, 2 for often, and 3 for always. The total score ranges from 0 to 30 points. The EPDS has been validated to assess depression in women and men in the perinatal period. Good sensitivity and specificity have been reported for the Chinese version of EPDS (Wang et al., 2009), with a cut-off score of 9/10 at postpartum, indicating the likely presence of depression. The scores of severe PPD were greater than 12 (Kheirabadi et al., 2012). The Cronbach alpha value of EPDS is 0.81.

In addition, due to different degrees of sleep problems and psychological stress in both groups, we used the Pittsburgh Sleep Quality Index (PSQI) to evaluate the sleep quality of the two groups. PSQI was compiled by Buysse and his colleagues in 1989, mainly used to evaluate the sleep quality of patients with sleep disorders and mental disorders, and used in studies related to PPD (Buysse et al., 1989; Zhu et al., 2018). The Cronbach alpha value of PSQI is 0.84. It consisted of seven items: sleep quality, fall asleep time, sleep efficiency, sleep disorder, hypnotic drugs, and daily time. Each item ranged from 0 to 3 points, and the total score was 21. The higher score indicates the worse sleep quality. A score of 0–5 indicates very good sleep, 6–10 normal sleep, 11–15 not good sleep, and 16–21 poor sleep. If the patient had more than seven points, they were deemed as having sleep disorder.

We also performed the Hamilton Anxiety Rating Scale (HAMA) and the Hamilton Depression Rating Scale (HAMD) to assess the severity of anxiety and depression (Hamilton, 1959, 1960; Lin et al., 2018; Li et al., 2019). The Cronbach alpha value of HAMA is 0.77. The Cronbach alpha value of HAMD is 0.79. HAMA evaluation criteria: scores less than seven indicates no anxiety; scores from 7 to 20 indicate likely to have anxiety; scores from 20 to 29 indicate certain anxiety; and scores above 29 are considered as severe anxiety. HAMD evaluation criteria: scores less than 8 indicates no depression; scores from 8 to 20 indicate likely to have depression; scores from 20 to 35 indicate depression; and scores above 35 indicate major depression.

In this double-blind trial, the patients in the two groups were not informed about their grouping during the HAMA, HAMD, EPDS, and PSQI assessments. These assessments were carried out at the 28 weeks of gestation, post-delivery (24 h postpartum) and post-intervention (6 weeks postpartum).

Nursing satisfaction was evaluated by the nursing satisfaction questionnaire developed by the department of obstetrics department in our hospital. There are 20 items in total and 1–5 points for each item. The total score is 100 points (scores ≥90 points indicate very satisfied; scores of 75 to <90 indicate satisfied; scores of 60 to <75 indicate basic satisfaction; and scores <60 indicate dissatisfied). It mainly includes four periods of prenatal examination, delivery, postpartum hospitalization, and post-discharge return visit. The survey content consists of the satisfaction of the environment, nursing staff’s attitude, the satisfaction of nursing staff’s methods, the satisfaction of nursing effect, and the overall satisfaction.

The Cronbach alpha value of EPDS is 0.81. The Cronbach alpha value of PSQI is 0.84. The Cronbach alpha value of HAMA is 0.77. The Cronbach alpha value of HAMD is 0.79. Calculated by SPSS, the Cronbach alpha value of our nursing satisfaction questionnaire was 0.72, which showed good reliability.

### Data Analysis

Statistical Package for the Social Sciences (SPSS) software was used to analyze our data. Comparisons between the two groups were performed by two-way ANOVA test followed by Tukey multiple comparisons test. To examine the distribution between categorical variables, we used a Chi-square test or Fisher’s exact test. *p* value less than 0.05 was considered as statistical significance.

## Results

### Characteristics and Satisfaction of the Patients

In our study, the basic characteristics of the control group and the SFM group were shown in Table 1. More than half of the patients chose standard vaginal delivery (63.2% in the control group and 71.2% in the SFM group). There was no significant difference observed between the two groups. Other characteristics, including age, abortion history, education, occupation, sex of the baby, and home support, showed no significant differences between the control and SFM groups either. The results also indicated that the two groups were well randomized.

**Table 1:** Characteristics of analyzed participants.

Demographic characteristics	Study group		*p* value
	Control group (*n* = 57)	SFM group (*n* = 59)	
Age (years)			
19–29	26 (45.6%)	24 (40.7%)	0.6073
30–35	20 (35.1%)	19 (32.2%)	
35 and over	11 (19.3%)	16 (27.1%)	
Delivery			
Vaginal delivery	36 (63.2%)	42 (71.2%)	0.3570
Caesarian section	21 (36.8%)	17 (28.8%)	
Abortion before			
0	32 (56.1%)	30 (50.8%)	0.2226
1	16 (28.1%)	14 (23.7%)	
2	5 (8.8%)	13 (22.1%)	
3 and more	4 (7.0%)	2 (3.4%)	
Education status			
Junior high school and below	12 (21.0%)	11 (18.6%)	0.7901
Senior high school	29 (50.9%)	28 (47.5%)	
College and above	16 (28.1%)	20 (33.9%)	
Occupation			
Farmer	5 (8.8%)	3 (5.1%)	0.7301
Individual business	9 (15.8%)	11 (18.6%)	
White-collar employees	19 (33.3%)	18 (30.5%)	
Teachers, doctors, scientists, and engineers	15 (26.3%)	20 (33.9%)	
Others	4 (7.0%)	5 (8.5%)	
Does not work	5 (8.8%)	2 (3.4%)	
The sex of baby			
Girl	29 (50.9%)	32 (54.2%)	0.7171
Boy	28 (49.1%)	27 (45.8%)	
Home supports for caring the baby			
Yes	42 (73.7%)	39 (66.1%)	0.3738
No	15 (26.3%)	20 (33.9%)	

Values were presented with *n* (percentage, %). *p* values for each group were derived from Chi-square test or Fisher’s exact test. SFM, solution-focused model.

After the intervention, our nursing satisfaction questionnaire was conducted to assess the patients’ satisfaction with our study. We found that the SFM could significantly improve nursing satisfaction (*p* = 0.0046; Table 2).

**Table 2:** Comparison of satisfaction of the care between the two groups.

	Study group		*p* value
	Control group (*n* = 57)	SFM group (*n* = 59)	
Very satisfied	14 (24.6%)	31 (52.5%)	0.0046
Satisfied	15 (26.3%)	16 (27.1%)	
Basically satisfied	19 (33.3%)	9 (15.3%)	
Not satisfied	9 (15.8%)	3 (5.1%)	

Values were presented with *n* (percentage, %). *p* values for each group were derived from Chi-square test or Fisher’s exact test. SFM, solution-focused model.

### Effects of SFM on Anxiety and PPD

**Figure 2:**
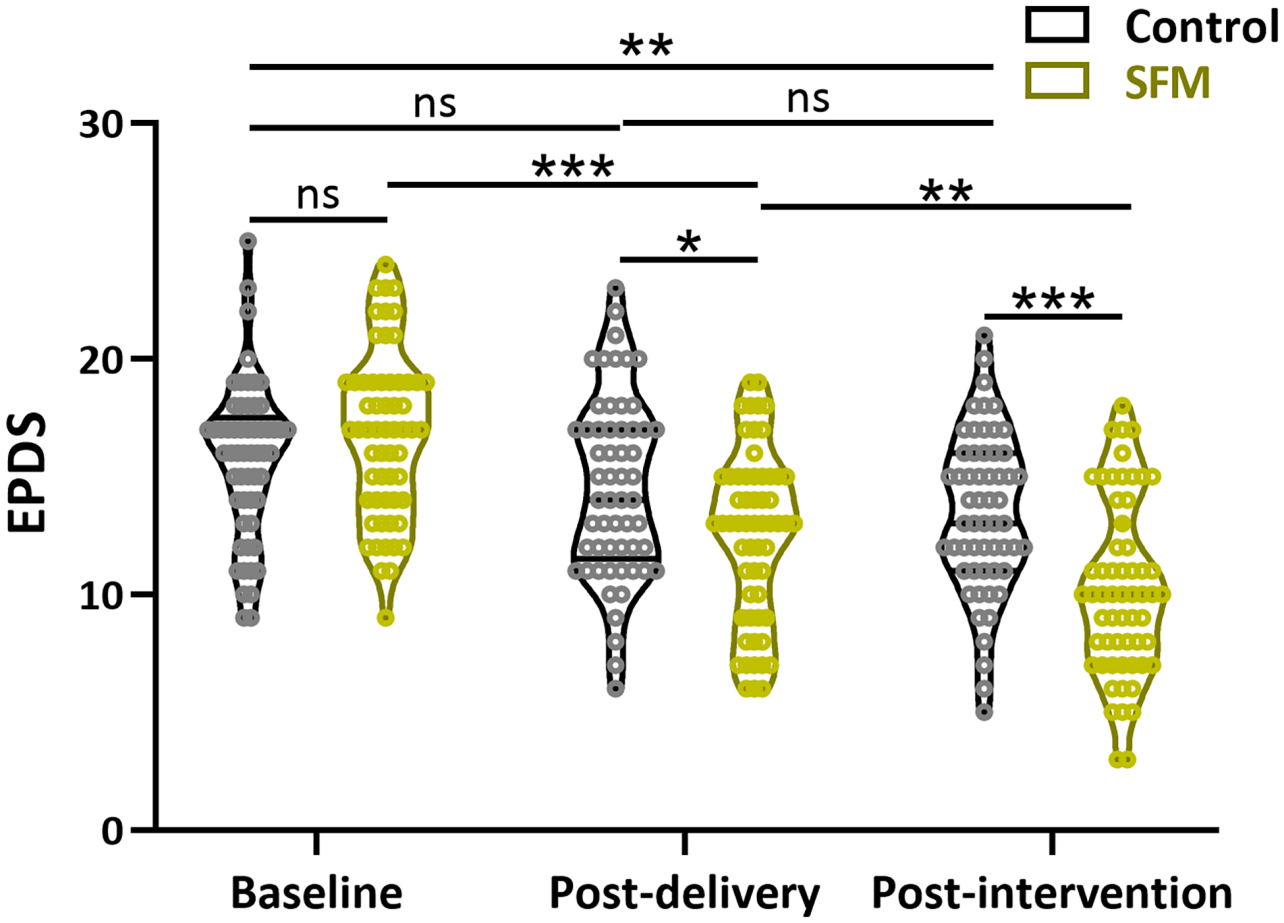
Assessment of Edinburgh Postpartum Depression Scale (EPDS) between the two groups at different time points. Values were presented with violin plot showing all the data. ^*^p < 0.05, ^**^p < 0.01, ^***^p < 0.001, and ns, no significance. Two-way ANOVA test followed by Turkey multiple comparisons test.

We used several assessments to evaluate the anxiety and depression levels of the patients at 28 weeks of gestation, post-delivery, and post-intervention. Table 3 compared the numbers of patients diagnosed with PPD by EPDS at the three different time points. The diagnostic criterion for severe PPD was greater than 13 points, which was used as our standard. There was no significant difference between the two groups at the beginning of the trial (28 weeks of gestation). However, there was a considerable difference between the two groups at post-delivery and post-intervention (*p* = 0.0366 and 0.0012, respectively). The total distribution of the assessment of EPDS was shown in Figure 2. The scores of EPDS in the SFM group tended to be less than those in the control group after intervention. These results indicated that SFM could reduce the rate of PPD more effectively.

**Table 3:** Comparison of frequency distribution of postpartum depression between the two groups at different time points.

Time points	Study group		*p* value
	Control group (*n* = 57)	SFM group (*n* = 59)	
Baseline	46 (80.7%)	49 (83.1%)	0.8120
Post-delivery	41 (71.9%)	31 (52.5%)	0.0366
Post-intervention	32 (56.1%)	15 (25.4%)	0.0012

Values were presented with *n* (percentage, %). *p* values for each group were derived from Chi-square test or Fisher’s exact test. Edinburgh Postnatal Depression Scale (EPDS) was used to diagnose the postpartum depression when the score ≥ 13. SFM, solution-focused model.

**Figure 3:**
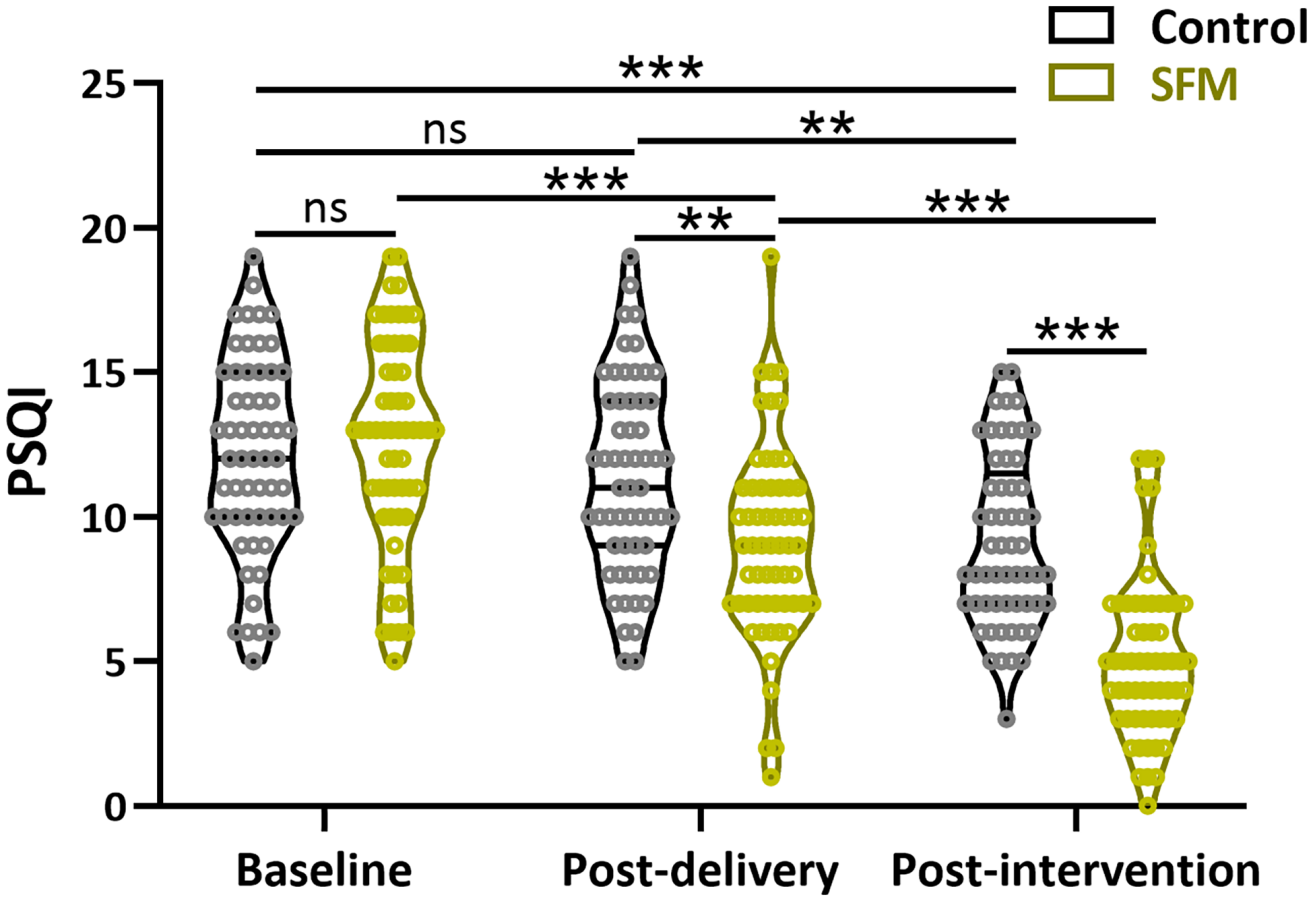
Assessment of Pittsburgh Sleep Quality Index (PSQI) between the two groups at different time points. Values were presented with violin plot showing all the data. ^**^p < 0.01, ^***^p < 0.001, and ns, no significance. Two-way ANOVA test followed by Turkey multiple comparisons test.

Then, we conducted a comparative analysis of PSQI scores between the two groups at the three different time points. A higher score stood for poorer sleep quality. A score less than seven points indicated sleep disorders. As shown in Figure 3, there was no significant difference between the SFM and control groups at the beginning of the trial. In contrast, the scores of PSQI in the SFM group were significantly less than those in the control group after intervention (*p* < 0.01), suggesting that sleep quality could be positively influenced by SFM intervention.

**Figure 4:**
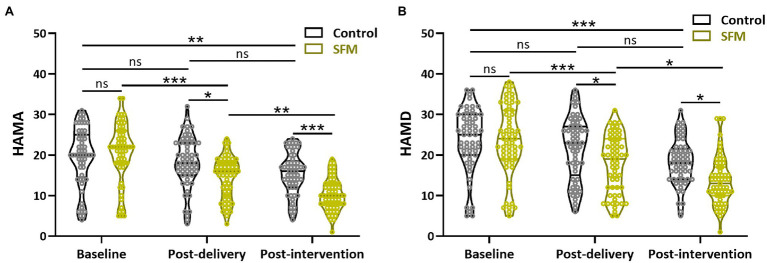
Assessment of Hamilton Anxiety Rating Scale (HAMA; **A**) and Hamilton Depression Rating Scale (HAMD; **B**) between the two groups at different time points. Values were presented with violin plot showing all the data. ^*^p < 0.05, ^**^p < 0.01, ^***^p < 0.001, and ns, no significance. Two-way ANOVA test followed by Turkey multiple comparisons test.

Lastly, the assessments of HAMA and HAMD between the two groups at three different time points were conducted. The result showed that the HAMA and HAMD scores at 28 weeks of gestation were not significantly different between the two groups (Figures 4A,B). However, significant differences between the two groups were observed at post-delivery and post-intervention (*p* < 0.05). Our results demonstrated that SFM could decrease the scores of anxiety and depression in our patients.

## Discussion

The pregnant and postpartum period is considered a time of vulnerability to affective disorder. PPD is one of the most common birth complications, usually occurring in 2–6 weeks after delivery. A previous study found that pregnancy anxiety and depression were mainly related to social, biological, and psychological factors (Karacam and Ancel, 2009). In addition, prenatal depression (Ho-Yen et al., 2007; Karacam and Ancel, 2009; Kirpinar et al., 2010; Ponti and Smorti, 2019) and anxiety (Liabsuetrakul et al., 2007; Raisanen et al., 2013; Ponti and Smorti, 2019) were reported to be closely related to the development of PPD. However, in China, the consultation rate was still low due to the insufficient attention to women with PPD from their families and the society.

There were a number of interventions for PPD. Compared with other interventions, the SFM focuses on success and strength and orients to the future. It could be applied to different clinical areas to improve patients’ life satisfaction and self-management ability. For example, during the COVID-19 global pandemic, the SFM was established in pediatric diabetes care and achieved positive clinical outcomes (Woodger et al., 2021). The SFM was also used in brain injury rehabilitation programs to help patients and their families (Gan, 2020; Finlayson et al., 2021). Furthermore, SFM was applied to cancer patients and their families, significantly improving patients’ life quality and relieving their negative emotions (Neilson-Clayton and Brownlee, 2002). Therefore, SFM might be a promising intervention for patients’ emotional problems. Our study tried to use SFM to intervene in the development of PDD and anxiety in the middle of the gestational period and investigate the effects of SFD on anxiety and PPD in nulliparous pregnant women compared to women with routine care services.

The SFM counselling typically consisted of five parts: describing the problem, developing well-formed goals, exploring exceptions, ending session feedback, and evaluating results. In our study, we investigated the effect of SFM counselling on pregnancy anxiety and PPD. We chose the mothers diagnosed as depressed or with a depressive tendency by EPDS at 28 weeks of gestation and divided them into the intervention and control groups. The mental resilience of nulliparous pregnant women is a dynamic changing process (Crane et al., 2019). Therefore, we chose three different time points: 28 weeks of gestation (base line), post-delivery, and post-intervention. The assessments (EPDS, PSQI, HAMA, and HAMD scales) were conducted at the three different time points to evaluate the anxiety and depression levels of the patients. Compared with the control group, our results showed that SFM decreased the scores of anxiety and depression more effectively and positively influenced sleep quality. By constantly excavating patients’ potential, SFM enabled them to alleviate and resolve their depression status through their resilience. We also found that SFM resulted in significantly higher nursing satisfaction than the control group (*p* = 0.0046). Therefore, it might suggest that SFM effectively prevented anxiety and PPD among nulliparous pregnant women. According to our results, SFM was a positive and desirable method to attenuate PPD. The current situation of insufficient attention to women with PPD leads to the low diagnosis rate of women with PPD. Therefore, SFM, as a non-passive approach, is more suitable for Chinese nulliparous pregnant women.

However, due to the small sample size of patients, the statistical power of our study was low. In addition, we did not conduct an intervention for the patients’ families, which would be expected to affect the intervention results. The counselling period was only 6 months, from 28 weeks of gestation to 6 weeks postpartum. We could extend the counselling period from the beginning of pregnancy in future study.

In conclusion, SFM could effectively alleviate anxiety and postpartum depression in nulliparous pregnant women. It could help mothers acquire abilities to prevent or relieve the symptoms of PPD and then improve the health of mothers and their families.

## Data Availability Statement

The raw data supporting the conclusions of this article will be made available by the authors, without undue reservation.

## Ethics Statement

The studies involving human participants were reviewed and approved by Shanghai Jiao Tong University Affiliated Sixth People’s Hospital. The patients/participants provided their written informed consent to participate in this study.

## Author Contributions

SH designed and supervised the study. CH and WH performed experiments and analysed data and wrote the manuscript and manuscript revisions. All authors contributed to the article and approved the submitted version.

## Funding

This research was supported by Shanghai Municipal Education Commission, Gaoyuan Nursing Grant Support (no. Hlgy1816ky).

## Conflict of Interest

The authors declare that the research was conducted in the absence of any commercial or financial relationships that could be construed as a potential conflict of interest.

## Publisher’s Note

All claims expressed in this article are solely those of the authors and do not necessarily represent those of their affiliated organizations, or those of the publisher, the editors and the reviewers. Any product that may be evaluated in this article, or claim that may be made by its manufacturer, is not guaranteed or endorsed by the publisher.
